# Temperature, Color and the Brain: An Externalist Reply to the Knowledge Argument

**DOI:** 10.1007/s13164-017-0358-z

**Published:** 2017-09-07

**Authors:** Paul Skokowski

**Affiliations:** St Edmund Hall, Oxford University, Queen’s Lane, Oxford, OX1 4AR United Kingdom

## Abstract

It is argued that the knowledge argument fails against externalist theories of mind. Enclosing Mary and cutting her off from some properties denies part of the physical world to Mary, which has the consequence of denying her certain kinds of physical knowledge. The externalist formulation of experience is shown to differ in vehicle, content, and causal role from the internalist version addressed by the knowledge argument, and is supported by results from neuroscience. This means that though the knowledge argument has some force against material internalists, it misses the mark entirely against externalist accounts.

Frank Jackson’s example of Mary and his knowledge argument against materialism are deservedly famous. Mary, a brilliant neuroscientist, has lived her entire life in a black and white room. She knows everything there is to know about neuroscience, indeed, she knows everything physical there is to know. She has learned all this by reading black and white books, watching lectures on black and white screens, and so on. However, despite the immense knowledge that Mary has, it seems that there is some knowledge that she lacks. For it seems intuitive that when she steps outside of the room for the first time, and is handed a red rose, she will, for the first time, *experience* red, and this experience will be a kind of knowledge that she heretofore lacked. But Mary already *had* all the physical knowledge, so this new knowledge is non-physical.

Here is a very simple, but general, form of the knowledge argument:

1. Mary has all the physical knowledge at time **t**.
2. Mary learns new knowledge at **t** + **dt**.

Therefore:

There is knowledge that escapes the materialist, and hence is nonphysical.

But there is a hidden assumption to this argument, which is simply stated:

(3) Mary can *have* all the physical knowledge while being confined to her room.

However, if this assumption (3) is false, then the argument fails. That is, if Mary can *not have all the physical knowledge* while being confined to her room, then knowledge she gains when leaving the room may be physical knowledge after all. As Jackson himself puts it, “What she knows beforehand [in her room] is ex hypothesi everything physical there is to know” (Jackson 1985, p. 292), which makes assumption (3) explicit. The arguments to follow may be taken as denying this assumption through, at least, failure to recognize an alternative formulation of materialism. The conclusion, then, does not follow.^1^

As I see it, this assumption is fair game to the materialist. And a particular type of materialist, namely an externalist, has the apparatus to show how this assumption can fail to hold for the kinds of knowledge at issue in the argument.^2^

For Jackson, having knowledge about some property, state, etc., means having the information about that property, state, process, etc. (Jackson 1982) Mary learns something about the world when she steps out of the room and experiences color for the first time.^3^ Experience or sensation delivers information from the senses - phenomenal information, or qualia (colors, tastes, etc.). According to Jackson, these “qualia are left out of the physicalist story.”(Jackson 1982, 130) Qualia, on this view, are not physical. Indeed, the Knowledge Argument’s strength, he claims, “…is that it is so hard to deny the central claim that one can have all the physical information *without having all the information there is to have*.”(Jackson 1982, 130) The conclusion of the knowledge argument is that experiential information - the very content of our experiences or qualia - is not physical information. I will call information or knowledge that is not physical (or ‘escapes the physical story’, as Jackson puts it), nonphysical information or nonphysical knowledge. (Jackson 1982, p. 130; Jackson 1985, p. 293) Following Jackson (1982, p. 129, 130) examples of this sort of information will include colors, tastes, sounds, and the bodily sensations, among others. These of course are qualia. I will reserve the label **E** for denoting experiences with this sort of content; experiences of qualia. Also, following Jackson, I will assume that we have knowledge that is not experiential in nature, for example, knowledge that 1 + 1 = 2, knowledge that Obama is president of the United States, or knowledge that Judith is in neural state X at time t. I will reserve the label **K** for denoting knowledge of this sort that is not experiential. Thus, physical information will not only be about particular properties and states, but also their interrelations, both in terms of tokens (for example the relation of this brain state to some external state – such as the state of a rose external to it), and also in terms of types, such as the way a scientist formulates physical laws (for example, this type of physical state will cause that type of physical state under these circumstances).^4^

The problem, as I see it, is that a materialist can see the physical limitations of Mary within her room as just that: physical limitations. By enclosing Mary physically, and cutting her off from direct experience of some physical properties of the world, Jackson denies some knowledge of the physical world to Mary.^5^ If one is a materialist of the internalist persuasion,^6^ this *can* pose a problem of the sort as Jackson envisions.^7^ For knowledge, although understood to be a physical thing for such a materialist, must be in the head. If Mary has all the physical knowledge, she must know about red before she exits the room, for all this knowledge is already *in her head*. But the intuition here is that Mary experiences something new, and so gains incremental, new, knowledge and information about colors when she exits the room. The internalist materialist is in a quandary: despite having all the physical knowledge of red inside her head within the room, she must now account for her new knowledge of red upon stepping outside.

However internalism is not the only position available to the materialist. If one is a materialist of the externalist persuasion, then one can hold that colors are properties of physical objects – where these objects are outside of the head. An externalist can maintain that certain kinds of physical knowledge – for all knowledge is physical to a materialist – are denied to Mary by the physical limitations to which she is peculiarly subjected. Thus, Mary’s physical limitations mean that she can *not* have all the physical knowledge while being confined to her room.^8^ The original assumption (3) therefore is false, or at the very least requires its own further justification, either of which is sufficient to make the Knowledge argument as a whole fail.

It should be noted from the outset – and this is the important point – that in order to defeat the knowledge argument, one does not need to actually establish materialism. All the materialist has to do is to show that there is a form of materialism that does not fall under the purview of the knowledge argument. And that is all that will be attempted in this paper. The goal here is to show how a form of materialism – what I shall call externalism – evades the net cast by the knowledge argument, and thereby shows its restricted applicability to internalist formulations of materialism.^9^

Let’s clear something right up front:

We know Mary learns something when she steps outside her room. Now, it is clear from the knowledge argument itself that Mary gains knowledge, as Jackson says “about the world and our visual experience of it.” (Jackson 1982, 130). Elsewhere, Jackson says Mary gains knowledge about other people. (Jackson 1985, 293) We have already been told that qualia of a certain sort (Red, say) have been left out of Mary’s physical knowledge. We know at a minimum therefore, that Mary gains knowledge that person X experiences quale R (Red) when seeing a red rose. This is Jackson’s point about Mary gaining knowledge about other people. But here is something we also know, that Mary gains the knowledge that *she* experiences R when seeing a red rose at t + dt.^10^ These are uncontroversial, since we know Mary learns something when she steps out of the room, and that what’s learned include those ‘…mental states which are said to have…qualia.’(Jackson, 1982 p. 130) Following standard usage I’m calling these mental states experiences or sensations.

Now the question comes up, are these experiences or sensations, such as the experience of (the quale) R, themselves a kind of knowledge? The latter seems plausible, for Mary is certainly experiencing Red for the first time. This is something new for her, at the very least a new experience of a new quale R. But, as we shall see in subsequent sections, experiential states differ from knowledge states in vehicle, content and causal role. I will deal with these differences in much more detail later, but one distinction we can mention now is that experiences are taken by externalists to be internal states that directly and causally co-vary with an external property. That property, therefore, is the content of the experience, and is not the sort of thing we would generally take to be the content of a that-clause; that is, that property is not understood to be propositional. Indeed, following Dretske (1990, 1995) and Crane (2012) on simple seeing, when Mary steps out of the room she is ‘seeing Red’ or ‘experiencing Red’ for the first time. This is a non-propositional mental state that is quite familiar in the philosophical and psychological sense^11^ (Crane 2012; Dretske 1990; Dretske 1995). So there is a technicality here: Must all knowledge that is discovered by Mary upon her exit be propositional?

I think it’s a natural intuition to say that when Mary experiences Red for the first time, that experiencing by itself is a kind of knowledge, and so counts as something Mary learns. But a purist on knowledge might reply that non-propositional knowledge just doesn’t count. Under this view, the experience of Red *simpliciter* isn’t knowledge, whereas Mary learning that she experiences Red at t + dt and her learning that other people have experienced Red will count as knowledge: knowledge that Mary experiences Red at t + dt, and knowledge that other people have experienced Red.

Reasonable people can disagree about whether an experience by itself counts as knowledge. I think it should count. But it will turn out that under either view, the knowledge argument fails against externalism. Let’s start by looking at temperature.

## Temperature

Mary is now back in her black and white room. But this room is a little different. It is always kept to 98.6 degrees. Any objects that are introduced to the room are at this temperature or higher. Mary likes hot coffee, and sometimes the coffee is so hot that it scalds her tongue. If she leaves her cup of coffee out, it eventually equilibrates to 98.6 degrees. Of course, though being restricted to her room, Mary knows everything there is to know about temperature, thermodynamics, and neuroscience.

Mary knows what it is like to feel 98.6 degrees. She feels it every day. After all, her room is in equilibrium, and her desk, chair, floor, walls, and many other objects in the room are constantly at that temperature. But she also knows what it is like to be 170 degrees, because that is what the temperature of her coffee is when she pours it fresh out of the pot and tastes it. She also knows what it is like to be 140 degrees, because that is the temperature her black and white computer reaches after being left on overnight. Indeed, we can assume that Mary knows quite a range of temperatures from 98.6 on up.

Mary is allowed to leave the room one day to an adjacent room. This room is also black and white, but it is nevertheless different – this room is kept at 30 degrees. What will happen when Mary enters this room? Following Jackson, ‘it seems just obvious that she will learn something’. Mary will learn what it is like to be COLD, in particular, what it is like to be 30 degrees. Thus her knowledge was incomplete. But she already had all the physical knowledge. So this is nonphysical knowledge.

Mary is a brilliant scientist. If so, it seems interesting that, with all she already knows about temperature, she is surprised to find that she has learned something, particularly something non-physical, upon stepping out of the room. Would the externalist (and Mary) agree?

Mary knows, for example, that we have specialized detectors – nerves at our extremities – which have the function of detecting temperatures. In fact, Mary holds that when she experiences the temperature of an object, *what* she experiences, the *content* of her experience, is the temperature of that object. She recognizes this as an instance of a physical law: When objects of type ***T*** are brought into contact with her extremities, then this will cause an event in her brain – neural firings in somatosensory cortex (Purves et al., 2001, Gazzaniga et al. 2002) – of type ***E***.

Mary knows that objects with temperatures (like 30 degrees F) and experiential states (such as a particular set of neurons firing in somatosensory cortex) are physical states. Their properties are instances of types, which physicists and neuroscientists would refer to as *Temperature* and *Sensory Neural Firing*. The types are connected in physical laws (Kim 1973). Particular instances of these physical laws are studied and verified in laboratories. It is known, for example, that when an object of temperature 400 degrees F is brought into contact with Mary’s hand, she will withdraw it. This temperature will cause the brain event, which will in turn cause motor neurons to fire, resulting in Mary rapidly withdrawing her hand from the object. Mary knows all this.

Mary also knows (as does the externalist) that when she experiences 400 degrees, nothing in her brain is 400 degrees. In particular, the state **E** in her somatosensory cortex is not 400 degrees. She recognizes that it is the external object that has that property.

Consider a completely material scientific theory of temperature and the sensing of temperature. Under such a view, temperatures are properties of objects, experiential states are properties of brains, and certain experiential states have the natural function of indicating temperatures. It is the latter’s job to indicate temperatures. This is a job conferred by natural selection. Under such a view, a particular experience of temperature involves a relation between the property of an external object and the property of an internal state. The externalist chooses this explanation as the best physical one on offer (Dretske 1995; Tye 1995, 2000).

Of course, nothing prevents Mary from holding such a view, so, to make things interesting, let’s suppose that Mary does. It is not essential to the arguments that follow that Mary holds such a view. But it is worth emphasizing that Mary can very well be a materialist and so fully expect to gain new, physical, knowledge upon opening the door. What makes this interesting in Mary’s case, is that in holding such a materialist view, then Mary already *expects* to learn something new when she steps out of her room. Mary, despite her vast knowledge, expects to gain the experience of a new property upon opening the door. She understands beforehand that she does not have all the physical knowledge while being confined to her room.

Now recall that such an experience, according to the knowledge argument, will give Mary new information, and ‘she will learn something about the world’. (Jackson 1982, p. 130) Mary knows the experience of 98.6 degrees and of 400 degrees because she has experienced them. The right covariational relationship exists for Mary such that an internal experiential state **E**, indicates, or carries information on the temperature **T**. Mary’s *experience* of temperature 400 degrees carries the *information* 400 degrees.(Dretske 1995) Let’s write this relationship in the form **E**(400). This is a shorthand for the particular token temperature ***T*** = 400 degrees causing the token neural state **E**, and for **E** carrying the information ***T*** = 400 degrees. For some arbitrary temperature **T** we can write, more generally, **E**(**T**).

When Mary steps out of the room she will experience 30 degrees, which exemplifies the relation **E**(30). Thus she gains information 30 degrees by directly experiencing that temperature. Being the content of a bodily sensation, Jackson says this new information is a quale, and so must be non-physical. The externalist agrees with Jackson that this is new information, but she disagrees that such information is non-physical.

For the externalist, all experience is physical. Both the experience *vehicle* – the internal neural state **E** – and the experiential *content* – the external physical property **T** – are physical. Further, the two are tied together by a nomic regularity, which underlies the ascription of informational content. But now note that if either the vehicle or the content is missing, then Mary does not have information. That is, having information requires *both* the vehicle *and* the content. I cannot have the information that the FedEx man is at my front door if the FedEx man is not at my front door. Here information is denied because the content is missing. Further, I cannot have the information the FedEx man is at my front door if I am at the beach without my cell phone, even if the FedEx man is at my front door. Here information is denied me because the vehicle is missing.

For the externalist, before Mary steps out the door, she lacks *both* the vehicle **E** *and* the content **T**. This means she cannot have the physical experience **E**(**T**). When she does step out the door she experiences the temperature 30 degrees. She then has the *physical experience* **E**(30), which was denied to her by her confinement. She is not having a physical experience of a non-physical content: 30 degrees is a material temperature – a physical property (Tolman 1956). 30 degrees is not a non-physical property, nor is it new non-physical information.

A counter to this would be, “Of course Mary can’t *experience* 30 degrees before stepping out of the room. But she can *know* about 30 degrees before stepping out of the room in other ways. After all, she’s been allowed to observe others who have experienced 30 degrees, and she has had access to fMRI scans of people experiencing 30 degrees, and so forth. So she has all the physical information about 30 degrees before leaving the room”.

The externalist agrees that Mary has a certain kind of knowledge before leaving the room. But, the externalist points out that this is a very different kind of knowledge – different in both content and causal role, and that the knowledge - *and experience* - at issue is denied to Mary through physical confinement.^12^ Let’s look at these points.

When Mary observes (through her black and white monitor) Bert experiencing 30 degrees, she can, the externalist agrees, have a certain kind of knowledge state. The kind of knowledge state Mary is in is a neural state, call it **K** ^**Mary**^ (where the superscript ‘Mary’ indicates this is Mary’s internal state), which has as its content Bert’s neural state which is his experiencing, call it **E** ^**Bert**^, and the temperature being experienced, 30 degrees, which is the informational content of Bert’s internal experiential state **E** ^**Bert**^. I will write Mary’s knowledge that Bert experiences 30 degrees as: **K** ^**Mary**^ [**E** ^**Bert**^ (30 degrees)]. This spells out that Mary’s internal state **K** ^**Mary**^ has the content **E** ^**Bert**^ (30 degrees) - that Mary knows that Bert is experiencing 30 degrees.

But Mary understands that her *knowledge* that Bert experiences 30 degrees is not the same as her own *experience* of 30 degrees. Mary is, after all, a learned neuroscientist. So she already knows that her knowledge states of the experiences of others are not the same as her own sensory experiences. Spelling it out in the terminology we have adopted, Mary knows that her knowledge state **K** ^**Mary**^ [**E** ^**Bert**^ (30 degrees)] ≠ **E** ^**Mary**^ (30 degrees). How could they be the same? It is not only that Mary and Bert are different individuals, and so they will have different vehicles instantiated in their respective heads (Mary’s internal neural state **K** ^**Mary**^ ≠ Bert’s internal neural state **E** ^**Bert**^), but also that the *contents* of the two types of knowledge are completely different. The content of Mary’s knowledge state is Bert experiencing 30 degrees, which involves a state in Bert’s brain. The content of Mary’s experience of 30 degrees is simply the temperature 30 degrees, which doesn’t involve Bert in the slightest. Both the vehicles *and* the contents differ.

But what Mary learns by stepping out the door is what it is to *experience* 30 degrees. And *this* learning includes an exemplification of the direct experience of that temperature, that is: **E** ^**Mary**^ (30 degrees). The externalist already understands that Mary will not have that vehicle and that content until *after* she steps out of the room. She also understands that Mary’s *knowledge* of 30 degrees through channels other than direct experience of that temperature – such as acquiring knowledge through her black and white monitor - is not the same as her *experience* of 30 degrees. The two vehicles **K** and **E** are, again, completely different neuronal states in the brain, with distinct locations and etiologies.^13^ Furthermore, Mary’s knowledge and experiential states are not simply different internal vehicle *tokens*, but also they are of different *types* altogether. The experiential type **E** is related to temperatures **T** only through a sensory modality, and a token experience like **E**(30 degrees) is caused directly by the external tokening of 30 degrees. In contrast, the knowledge type **K** is installed through time from a learning history, and (the internal state) **K** is *not* caused directly by a token **T** such as 30 degrees, but rather by a history of Mary’s interactions with instructors who talk about **T**, with instruments that measure **T**, textbooks that show blackbody curves for **T**, and so forth.^14^ These two kinds of states (knowledge and experience) are exemplified in different regions of the brain, and are caused in completely different ways.

Further, the two types of internal vehicles have different causal consequences. One causes withdrawal of the hands, and the other doesn’t – rather, the latter type typically causes further thoughts or statements (‘has a blackbody shape with peak at …’). The externalist, and a neuroscientist such as Mary, knows that the two state types **E** and **K** occur in different parts of the brain. Therefore both the vehicles, and their causal roles – both the kinds of events which cause these vehicles and the kinds of events which issue from them – differ.

One could reply here that because Mary has knowledge of type **K** about 30 degrees before she steps out of the room, then she already has all the physical knowledge. But the externalist (and Mary) begs to disagree. She can point out that Mary already has knowledge – and experience – of other instances of temperature, for example, 98.6 degrees and 400 degrees. Furthermore, she understands that the *experience* of these temperatures is different in type from *knowledge* of these temperatures: an example of the latter would be Mary thinking of the blackbody curve for the temperature 120 degrees. She might even say, “When I experience 120 degrees, it *feels* differently from my knowledge states of that temperature. But, as a neuroscientist, I understand that when I directly *feel* 120 degrees, my somatosensory cortex is stimulated from detectors on my periphery, whereas when I *think* of 120 degrees, another set of neurons entirely is firing. I therefore understand that the two types of states – the experiential state **E**, and the thinking (knowledge) state **K**, *are* different, both in their physical vehicles and in their causal relations with other physical states. I therefore understand that my experience of 30 degrees when I step out of the door will differ from my knowledge of 30 degrees, in a similar way that other experiences of temperatures differ from knowledge states of those temperatures. However, the physical experience of 30 degrees, **E**(30 degrees) is denied to me by the physical configuration I am in. This configuration denies me the physical vehicle for the experience, and the physical causal relations required to exemplify that state in my brain. I am therefore being denied *this* kind of physical knowledge and experience.” Thus, though Mary by hypothesis knows (**K**) about 30 degrees, she also knows that she lacks the experiential relation **E** (30 degrees). In addition she realizes she lacks direct knowledge of this experience **K** ^**Mary**^ [**E** ^**Mary**^ (30 degrees)], for this state also can’t be instantiated until after she steps out of the room.^15^ So *some* physical knowledge and experience has, after all, been left out of Mary’s surroundings by virtue of her confinement. She has not, as assumed, been given *all* the physical information.

## Color

The argument just made about temperature can be carried over mutatis mutandis to color. Jackson himself recognized this (in reverse) when he said “the same style of Knowledge argument could be deployed for taste, hearing, the bodily sensations…” (Jackson 1982, 130) Here is how it works.

Mary is in a black and white room. I will assume that since Mary watches black and white TV then she also sees grays. It would be difficult to design a TV that had the fidelity necessary to convey experimental apparati, or fMRI scans (which express activation levels in the brain via differences in shading) to Mary without shades of gray. Also, shadows in her room would appear as shades of gray. So I will assume that Mary is familiar with some shades of gray between black and white.

Now, it might not seem that black and white and gray are colors, yet a good many color scientists (and philosophers) consider them to be so. In well-lighted rooms, we see white, black, and shades of gray. But our rods, which carry only luminance information, are fully saturated in such light, and so are not delivering information on these shades - only the cones are functioning fully. So, if Mary is in a room with only black, white, and gray, she is experiencing these colors by virtue of the very same apparatus (her cones) that mediates color vision.

Now recall the example above about temperature. Mary had the sensory apparatus to deliver information on a wide range of temperatures. However, because she was kept within a certain temperature range when confined to her room, she was unable to experience certain temperatures. The same thing is happening to Mary now with respect to the chromatic colors (blue, red, yellow, etc.). The externalist considers all the colors, achromatic (white, black, grey) and the chromatic colors (blue, red, yellow, etc.) to be properties of external objects, for example, reflectances (Dretske 1995, Byrne and Hilbert 2003, Hilbert 1987, Tye 1995, 2000).

With regard to sensing temperatures, Mary was previously allowed to experience only a limited temperature range of 98.6 degrees to 400 degrees. Then she was allowed to move out of the room. This gave Mary access to other physical properties and the causal relations which mediate their interactions with her sensory systems – in particular a new temperature range of 98.5 degrees and below.

With regard to sensing colors, Mary was previously allowed to experience only a limited reflectance range of white, grays, and black. Now she is allowed to move out of the room. This gives Mary access to other physical properties and the causal relations which mediate their interactions with her sensory systems – in particular a new reflectance range which now includes the chromatic color reflectances.

As was the case for temperatures, Mary knows a lot about colors and color perception. She has knowledge **K** about the reflectance for red, call it **R**, but, as was the case for temperatures, she fully expects that her *knowledge* (through channels other than direct experience of the color red – such as acquiring knowledge through her black and white television) of the reflectance for red will not be the same as her direct *experience* of that reflectance. Thus **K**(**R**) ≠ **E**(**R**). The reason she knows this is that she has *already* experienced colors – black, white and gray. These color experiences have been delivered by the same sensory system – mediated by the cones – that will deliver colors like red. Mary *knows* what an *experience* of a color is like. And she understands that the experience of a new color will be different, because it will link a different external property, for example **R**, with a different internal vehicle, **E**.

She understands this because her previous direct experiences of external color properties already differed in feel from her knowledge states about those properties as reflectances. She knows that experiencing white is different from knowledge of white’s reflectance (call it **W**). And she knows why this occurs. When Mary experiences white, she undergoes the experience **E**(**W**), where the experiential state, as she knows, is a neural state in her visual cortex, and white, **W**, is a property of an external object. Similarly for black, and for shades of gray. When she thinks about the reflectance curve for white, she has the knowledge state **K**(**W**), which, as she knows, does not occur in visual cortex, but in another part of her brain. She knows, for example, that the regions activated in *knowledge* of (as opposed to direct perceptual *experience* of) colors include left frontal cortex, left inferior temporal, left posterior parietal and other areas (Martin et al. 1995; D’Esposito et al. 1997; Miceli et al. 2001; Chao and Martin 1999; Wiggs et al. 1999.) Further, she knows that these areas are separate and distinct from visual cortex, which is the locus of direct visual experience of color. (Martin et al. 1995; D’Esposito et al. 1997; Miceli et al. 2001; Chao and Martin 1999; Noppeney and Price 2003, Wiggs et al. 1999; Zeki, 1993.) As was the case with temperatures, Mary understands that different vehicles for information will feel different. But this is as it should be, since the neural states themselves differ in physical properties (different neurons with a different physical locations in the brain) and have fundamentally different causal origins and causal consequences: historical causes for **K**, and direct covariational causes for **E**; thinking of finer details of reflectance curves after thinking **K**(**W**), and reaching for white chess pieces when in state **E**(**W**). Why *should* they feel the same?

Further, Mary knows that color experiences are different in kind from knowledge states in that experiences are direct, unmediated sensations of color. Mary knows what it is like to experience color – she experiences blacks, whites, grays. Color properties sensed through visual channels by her cones yield color experiences. So she understands that when she experiences red for the first time, this experience will differ from her knowledge of red. Again, the vehicles **K** and **E** are completely different – experience vehicles of red occur in visual cortex while knowledge vehicles of red reflectance occur in frontal cortex. Further their causal roles (both the kinds of events which cause these vehicles and the kinds of events which issue from them) differ: historical causes for **K**, and direct covariational causes for **E**.

## Thinking about Experience, or Rejecting the Assumption

The externalist formulation of materialism is one in which Mary will acquire new, physical, knowledge when she steps out of the room. The externalist therefore denies that Mary can have all the physical knowledge *while being confined to her room*. For confining her in this way limits the physical knowledge that Mary can have. The promise made by the knowledge argument that Mary can have all the physical information under these conditions is a disingenuous one.

It is interesting that Jackson later says that Mary will, upon her release, “realize how impoverished her conception of the mental life of others has been all along.”(Jackson 1985, p. 292) The interesting thing about this quote is that Mary, a competent neuroscientist, already realizes her impoverishment before stepping out of the room, from examination of her own case. Because she already realizes this from her own case, then *of course* she realizes it about others. She realizes that *thinking* – and here I mean the state **K** – about some property is completely different from *experiencing* that property directly through the senses. That is, thinking of a temperature of 189 degrees is completely different from experiencing 189 degrees, and thinking of a shade of gray is completely different from experiencing that shade. She can easily run the tests on herself for those ranges of properties she is allowed contact with in her room. Thus, she can think hard about 189 degrees, and compare that thought with the actual experience of sticking her index finger in her newly poured cup of tea. Mary knows these are different. She might even study her data and realize that she hasn’t been exposed yet to any temperatures between 180 degrees and 200 degrees. So she thinks really hard about these temperatures, and in particular, thinks about 189 degrees. And she understands that thinking in this way just is not like experiencing 189 degrees. She can even run a test and confirm this while still in her original room. She can turn on the black and white fMRI machine which is in her room, insert her head, and watch the internal black and white screen as one part of her brain lights up while she thinks (**K**) about 189 degrees, and then sees another part of her brain – somatosensory cortex – light up when she experiences 189 degrees by sticking her finger into the heated water. She confirms to herself that the two states feel completely different, and have different physical causes and effects. It just doesn’t follow that because Mary hasn’t experienced 189 degrees as of time **t** and then does experience 189 degrees at time **t** + **dt** that she thereby gains nonphysical knowledge from the experience. What does follow is that Mary has gained new *physical* information from a new experience - to wit **E**(189 degrees).

## Conclusion

We have seen that by enclosing Mary physically, and thereby cutting her off from direct access to some physical properties of the world, Jackson denies part of the physical world to Mary. This has the consequence of denying certain kinds of knowledge – experiential knowledge – of these properties.

Returning now to the discussion of knowledge and experience, we saw that there were two positions: denying experience as knowledge, or accepting experience as knowledge. Even if we do not count experience itself as a kind of knowledge, Mary gains knowledge when stepping out of her room that is parasitic on her new experience of a physical property, namely: knowledge that Mary experiences the property Red at **t + dt**, and knowledge that other people have experienced this property Red as Mary now has. But this new knowledge is physical knowledge that requires Mary to have experiential contact with a physical property that she has hitherto been denied by her physical configuration. As Crane puts it, Mary could not have known either truth in her black and white room ‘not because it is a truth about some mysterious non-physical feature of the world, but because it is the kind of truth that requires the knower to have an experience.’(Crane 2012, 197)

If, on the other hand, we do decide to count experience as a kind of knowledge, then Mary will immediately gain new physical knowledge when she steps out of her room: experiential knowledge. For the externalist, knowledge - and experience - are relations: material relations. Experience includes an internal experiential state, an external property (such as temperature or color) that is experienced, and a direct causal connection between the state and the property. Experience, or - if we accept it as such - experiential knowledge, therefore extends beyond the experiential state in the brain, and includes properties and regularities in the world external to its location in physical space.

By enclosing Mary physically, we cut her off from direct access to these physical properties in the world. By inserting a barrier between Mary and the world, and thereby breaking the physical relation necessary for experiencing new physical properties outside her room, Mary is denied certain kinds of physical experiential knowledge altogether.

For the externalist, physical barriers like these will always limit Mary’s knowledge, because experience is an extended thing. Thus assumption (1) of the knowledge argument - that Mary has all the physical knowledge at time **t** - is seen by the externalist as simply false. It is false because it relies itself on the further hidden assumption (3) that Mary can have all the physical knowledge while being confined to her room. By construction, this configuration already denies physical knowledge to Mary and so externalism rejects the assumption. Showing how this assumption applies to externalism requires a different argument altogether, which the knowledge argument simply does not provide. As I said in the beginning, the knowledge argument has some force against material internalists. But it misses the mark entirely against externalism.

## Footnotes

1 It should be noted that Jackson (2003, 2007) has recently joined the materialist camp.

2 I will follow Jackson (2003) in using materialism and physicalism interchangeably, and in construing materialism about mind as holding that “…the mind is a purely physical part of a purely physical world.” (Jackson 2003, p. 251)

3 In a sense I agree with Alter (2013): Mary’s new knowledge at t + dt (he calls it *knowledge*_*M*_) requires what he terms ‘mastery’. Following Alter, Mary makes epistemic progress at t + dt, and thereby obtains new *phenomenal knowledge*_*M*_ in addition to *knowledge*_*M*_ (Alter p. 492), despite Ball’s (2009) and Tye’s (2009) claims to the contrary. However, I disagree with Alter’s conclusion that this preserves the knowledge argument, as it is this very epistemic progress that shows how the knowledge argument fails against externalism.

4 Following, for example Goldman (1970), Mackie (1974), and Kim (1973) and their type/token formulations of events and causation.

5 The arguments in the sections on temperature and color that follow show how Mary is indeed cut off from forms of knowledge of qualia through her confinement.

6 For an internalist, knowledge is contained entirely in the head. For example, for an Identity Theorist internalist, knowledge states are identical with brain states, and so all knowledge is contained within the brain. (Place, 1956; Smart 1959)

7 Perry (2001), to give one example, gives a strong case for saving internalist-style materialism from Jackson’s argument.

8 There are others who have argued to the effect that Mary does not have all the physical knowledge (see, for example, Stoljar 2001 and Horgan 1984), but their arguments are not from the viewpoint of externalism, as defended here.

9 For the externalist materialist, the contents of mental states are generally located outside the head. See for example, Dretske (1996), Tye (1995), Skokowski (2007).

10 Tye (2009, 133) and Crane (2012, 196) put it a little differently, saying Mary learns ‘that this is what it is like to experience red.’ Either usage works for the purposes of this paper, and either will, once the arguments in the following sections go through, count as additional physical knowledge that Mary gains upon exiting the room.

11 I agree with Crane (2012) that this familiar notion of seeing can be used to address the knowledge argument without any need to invoke a Russellian notion of acquaintance. An externalist account of experience (including ‘seeing’) and knowledge will, in itself, serve to show how the knowledge argument falls short against materialist externalism.

12 This point is also made by Alter (2013, 492): Mary “…cannot arrive at the relevant phenomenal knowledge_M_ by deducing truths from her knowledge_M_ of the complete physical truth and her knowledge_P_ of phenomenal truths about what it is like to see in colour.” (Where knowledge_P_ is the knowledge she has beforehand in her room of others’ color experiences.)

13 See the next section for imaging studies that show separate neural correlates for knowledge and experience for a different sensory modality.

14 The role of learning histories for installing cognitive states is discussed in Dretske (1988). The differences for histories of cognitive and experiential states is discussed in Dretske (1995). Examples for installing cognitive states in neural substrates through learning are given in Skokowski (2004).

15 As mentioned above, Alter (2013) calls these new increments to Mary’s knowledge *phenomenal knowledge*_*M*_.
